# Associated factors for stress ulcers and gastrointestinal bleeding in patients with sepsis or septic shock: A systematic review

**DOI:** 10.1371/journal.pone.0361608

**Published:** 2026-10-30

**Authors:** Jin Wang, Xingwei Zhu, Linhui Jin, Yafei Liu, Man Tao, Zhijuan He, Xiaoxia Tang, Guilan Zhang, Hailin Zhu

**Affiliations:** 1 School of Nursing, Yunnan University of Traditional Chinese Medicine, Kunming, Yunnan, China; 2 Department of Neurology, Chongqing Hospital of The Affiliated Hospital of Guangzhou University of Chinese Medicine, Chongqing, China; 3 Department of Rehabilitation Medicine, Kunming Municipal Hospital of Traditional Chinese Medicine, Kunming, Yunnan, China; 4 Department of Critical Care Medicine, Kunming Municipal Hospital of Traditional Chinese Medicine, Kunming, Yunnan, China; 5 Department of Nursing, Kunming Municipal Hospital of Traditional Chinese Medicine, Kunming, Yunnan, China; Independent Medical Researcher and Writer, UNITED KINGDOM OF GREAT BRITAIN AND NORTHERN IRELAND

## Abstract

**Background:**

Sepsis continues to be a major contributor to death among critically ill individuals and places a heavy strain on healthcare resources. In addition to causing systemic inflammation and affecting hemodynamics, sepsis has a significant effect on the gastrointestinal system, leading to complications like stress ulcers and notable gastrointestinal bleeding (GIB), which worsen patient outcomes. While stress ulcer prophylaxis (SUP) has traditionally been included in sepsis treatment protocols, the concept of applying it universally is being increasingly scrutinized due to the absence of definitive mortality advantages and the risk of adverse effects, such as hospital-acquired pneumonia. Nevertheless, there is a notable gap in synthesized research that specifically addresses the independent risk factors for stress ulcers in sepsis patients.

**Methods:**

We carried out a systematic review in accordance with PRISMA standards, exploring eight databases (CNKI, Wanfang, VIP, Sinomed, PubMed, Web of Science, Embase, and Cochrane Library) from their inception until September 1, 2026. We included observational analytical studies (either cohort or case-control) that examined factors linked to stress ulcers or significant gastrointestinal bleeding in adult patients with sepsis. Two researchers independently handled the selection of studies, extraction of data, and evaluation of quality using the Newcastle-Ottawa Scale. Due to the limited number of studies for each association between associated factors and outcomes, a narrative synthesis was utilized.

**Results:**

A total of six studies encompassing 131,568 individuals suffering from sepsis or septic shock were analyzed. The incidence rates of stress ulcers or gastrointestinal bleeding (GIB) varied between 2.0% and 32.3%. The identified associated factors were divided into three categories: patient-related (older age, history of GIB, cirrhosis, HIV, cancer/immunosuppression), disease-related (coagulopathy, kidney impairment, high BUN levels, severity of illness scores, source of infection), and treatment-related (mechanical ventilation, use of anticoagulants, corticosteroid therapy, duration of ICU stay). Specifically for stress ulcer, factors such as respiratory failure, high BUN levels, shock, and increased HMGB1/TLR2/TLR4 levels were found to be independently linked to a higher odds.

**Conclusions:**

In summary, the likelihood of developing stress ulcers and gastrointestinal bleeding in patients with sepsis is shaped by a variety of factors related to the patient, their illness, and the treatments they receive. Key indicators that consistently emerge include issues with blood coagulation, the presence of shock, the need for mechanical ventilation, liver and kidney dysfunction, the severity of the disease, and any previous occurrences of gastrointestinal bleeding. These results advocate for tailored strategies for stress ulcer prophylaxis based on individual risk rather than a one-size-fits-all approach, offering crucial insights for precise clinical decision-making. Additional high-quality research is essential to confirm and enhance the understanding of these associated factors.

**Registration:**

PROSPERO (CRD420251242849)

## Introduction

Sepsis is characterized by an uncontrolled inflammatory reaction from the host in response to an infection [1]. It leads to approximately 50 million fatalities globally each year and poses a significant challenge to public health, being a primary cause of death among critically ill individuals, which places considerable strain on healthcare resources [2–4]. In addition to causing systemic inflammation and hemodynamic instability, sepsis significantly affects the gastrointestinal system [5,6]. The gut is not simply a passive entity suffering from reduced blood flow; it can also contribute to the failure of multiple organs through processes like barrier breakdown and the movement of bacteria. In this regard, stress-related ulcers and notable gastrointestinal bleeding are critical complications that increase patient morbidity and complicate treatment strategies [7].

As a result, the use of stress ulcer prophylaxis (SUP) has been a standard part of sepsis treatment protocols for some time [8]. Nevertheless, the concept of applying it universally is facing increasing scrutiny. Recent studies indicate that routine SUP may not provide a significant mortality advantage for all sepsis patients and point out possible adverse effects, including a higher likelihood of hospital-acquired pneumonia [9,10]. This underscores the critical need for a tailored medicine strategy to pinpoint those patients who are most at risk, thereby enhancing the benefit-risk balance of SUP. However, effective risk assessment necessitates a thorough understanding of the specific patient characteristics that can independently forecast the development of stress ulcers.

Numerous risk factors associated with gastrointestinal bleeding in critically ill patients, such as coagulopathy, mechanical ventilation, and shock [11], are acknowledged; however, their specific importance and impact within the sepsis population are not well understood. The diverse pathophysiological mechanisms of sepsis may influence these risks in various ways. Additionally, while these factors are often studied concerning significant clinical bleeding, their ability to independently forecast the onset of stress ulcers has not been thoroughly investigated. Importantly, there is a significant lack of consolidated research that focuses solely on the independent risk factors for stress ulcers in individuals suffering from sepsis and septic shock. This gap in knowledge may lead to excessive prophylactic measures in those at low risk, while failing to adequately protect patients who are at higher risk.

To enhance the development of focused and evidence-driven clinical approaches, this research will perform a systematic review. Our objective is to aggregate and evaluate the findings from observational studies regarding the factors associated with stress ulcers and gastrointestinal bleeding in adult patients suffering from sepsis. We seek to identify the patient-specific characteristics that most reliably forecast this complication within this unique, high-risk population, offering a thorough narrative and qualitative analysis.

## Methods

### Protocol and registration

The research plan was preemptively logged with PROSPERO (registration ID CRD420251242849). You can view the registered plan at this link: https://www.crd.york.ac.uk/PROSPERO/display_record.php?RecordID=1242849. This review adhered to the guidelines set forth by the Preferred Reporting Items for Systematic Reviews and Meta-Analyses (PRISMA) statement [12].

### Search strategy

We performed a thorough review aimed at identifying research related to the factors linked to stress ulcers and gastrointestinal bleeding in sepsis patients. Our search encompassed several databases, including CNKI, Wanfang, VIP, Sinomed, PubMed, Web of Science, Embase (Elsevier), and the Cochrane Library, covering all available literature up to September 1, 2026. The search utilized a combination of MeSH and free-text terms relevant to sepsis and gastrointestinal bleeding. Furthermore, we implemented the validated aetiology filter from the McMaster University Hedges Project, which enhances both sensitivity and specificity by including synonymous terms like ‘predictors’ and ‘associated risk,’ while reducing irrelevant findings. A comprehensive outline of our search strategy can be found in Table 1. We also manually reviewed the reference lists of all studies included to discover any additional pertinent articles.

**Table 1:** PubMed search strategy.

search	query
#1	((((((((((((((((“Sepsis”[Mesh]) OR (Bloodstream Infection[Title/Abstract])) OR (Bloodstream Infections[Title/Abstract])) OR (Infection, Bloodstream[Title/Abstract])) OR (Septicemia[Title/Abstract])) OR (Septicemias[Title/Abstract])) OR (Blood Poisoning[Title/Abstract])) OR (Blood Poisonings[Title/Abstract])) OR (Poisonings, Blood[Title/Abstract])) OR (Poisoning, Blood[Title/Abstract])) OR (Severe Sepsis[Title/Abstract])) OR (Sepsis, Severe[Title/Abstract])) OR (Pyemia[Title/Abstract])) OR (Pyemias[Title/Abstract])) OR (Pyaemia[Title/Abstract])) OR (Pyohemia[Title/Abstract])) OR (Pyohemias[Title/Abstract])
#2	“stress ulcer”[Title/Abstract] OR “gastric mucosal lesions”[Title/Abstract] OR “gastric mucosal erosion”[Title/Abstract] OR “gastric mucosa injury”[Title/Abstract] OR “stress related mucosal disease”[Title/Abstract] OR “gastrointestinal bleeding”[Title/Abstract] OR “gastrointestinal hemorrhage”[Title/Abstract] OR “upper gastrointestinal bleeding”[Title/Abstract]
#3	risk[Title/Abstract] OR risk[MeSH:noexp] OR mortality[Title/Abstract] OR mortality[MeSH:noexp] OR cohort[Title/Abstract]
#4	#1 AND #2 AND #3

### Inclusion criteria

We will evaluated studies for inclusion based on the following criteria: 1) Population: Adult patients (aged 18 years and older) diagnosed with sepsis or septic shock, as defined by sepsis-3 [13] or as outlined in the primary research. 2) Exposure & comparison: The study must explore one or more potential factors associated with the presence of stress ulcers or clinically significant gastrointestinal bleeding, including demographic, clinical, laboratory, or therapeutic factors. It should present data illustrating the relationship between these associated factors and the outcomes. 3) Outcome: The research must report the presence of stress ulcers or significant gastrointestinal bleeding. The criteria for diagnosing stress ulcers include: symptoms or signs indicative of upper gastrointestinal bleeding or abnormal lab results, such as: ① vomiting blood or fresh blood/brown fluid in nasogastric suction; ② a positive result on a gastric occult blood test; ③ melena or a positive fecal occult blood test; ④ findings from gastroscopy indicating a gastric ulcer or erosions. Clinically significant gastrointestinal bleeding is defined as visible bleeding that leads to hemodynamic changes necessitating a blood transfusion or endoscopic treatment. 4) Study design: The studies must be observational and analytical, encompassing both prospective and retrospective cohort studies as well as case-control studies.

### Exclusion criteria

We did not consider studies for the following reasons: 1) Type and format of publication: case reports, case series, narrative reviews, commentaries, editorials, conference abstracts lacking full data access, and studies conducted in vitro or on animals. 2) Study population: Research that focuses solely on children, pregnant individuals, or patients whose primary gastrointestinal issues (such as variceal bleeding or peptic ulcer disease at the time of admission) are the main cause of bleeding, as their pathophysiology and risk factors are significantly different. 3) Data and reporting standards: Studies that fail to present any descriptive, qualitative, or basic quantitative information regarding the relationship between a risk factor and the outcome within the sepsis study group.

### Study selection

Two researchers conducted the literature review, evaluation, and quality analysis separately. Initially, all collected records were uploaded into Endnote reference management software to eliminate duplicates. They proceeded to examine titles and abstracts to pinpoint studies that might meet the eligibility criteria. Following this preliminary review, the full-text articles of the chosen records were obtained and thoroughly evaluated for final selection. Any differences in opinion between the two researchers were addressed by involving a third researcher.

### Quality appraisal

The quality of methodology and potential bias in the cohort and case-control studies included in the review were evaluated independently by two reviewers utilizing the Newcastle-Ottawa Scale (NOS) [14]. This scale assesses studies based on three criteria: (1) the selection process of study groups, (2) the comparability between groups, and (3) the determination of exposure (for case-control studies) or outcomes (for cohort studies). A total of nine stars is the highest possible score across these criteria. Each study underwent independent evaluation by two reviewers, with any discrepancies addressed through discussion or by consulting a third reviewer. The studies were classified into three quality categories: high quality (≥7 stars), moderate quality (5–6 stars), and low quality (≤4 stars) for further evidence interpretation.

### Data extraction

For each included study, one researcher extracted the following data using a data extraction form: authors’ names, publication year, study location, design, characteristics of the study population, all examined associated factors, and the reported relationship of each associated factor with the outcome. A second researcher independently verified all extracted data. Any discrepancies were resolved through discussion, and a third researcher was consulted when necessary.

### Data synthesis

Given the scarcity of research on each specific associated factor and its corresponding outcome, conducting a meta-analysis was deemed unsuitable and lacking statistical significance. Consequently, a narrative synthesis method was utilized to encapsulate and analyze the results. Information regarding related factors and their adjusted effect sizes was gathered and organized into tables. The results were narratively synthesized by categorizing the related factors into clinically significant domains, thereby offering a coherent qualitative overview.

### Ethical consideration

This review synthesizes findings from existing literature, which means that it did not involve gathering new data or interacting directly with patients; thus, there was no need for further ethical clearance. However, we ensured that every study included in our review had received the necessary ethical approval from their relevant institutional review boards or ethics committees, or had an IRB exemption for research utilizing anonymized public databases, along with obtaining informed consent from participants when required.

### Data Availability Statement

#### Patient and public involvement

This systematic review did not involve any patients or public participants in its design, execution, reporting, or dissemination strategies, as it relies on data that has already been published.

## Results

### Search results

Fig 1 depicts the process and outcomes of literature selection. An initial search across various databases, including CNKI, Wanfang, VIP, Sinomed, PubMed, Web of Science, Embase (Elsevier), and the Cochrane Library, yielded 2,414 articles. Additionally, three more articles were found through reference tracking. After eliminating 644 duplicates, 1,770 articles were left for initial evaluation. Out of these, 925 were requested for retrieval, but 2 could not be accessed. The remaining 923 were evaluated for their eligibility. During this evaluation, 845 secondary studies (such as systematic reviews, meta-analyses, and reviews) were excluded, along with 803 articles that had discrepancies in research content, 88 due to issues with study design, 24 because of inconsistencies in outcome measures, and 5 because their statistical methods or results were insufficiently reported to allow assessment of the associations of interest. Ultimately, six studies were included in the systematic review [15–20].

**Fig 1:**
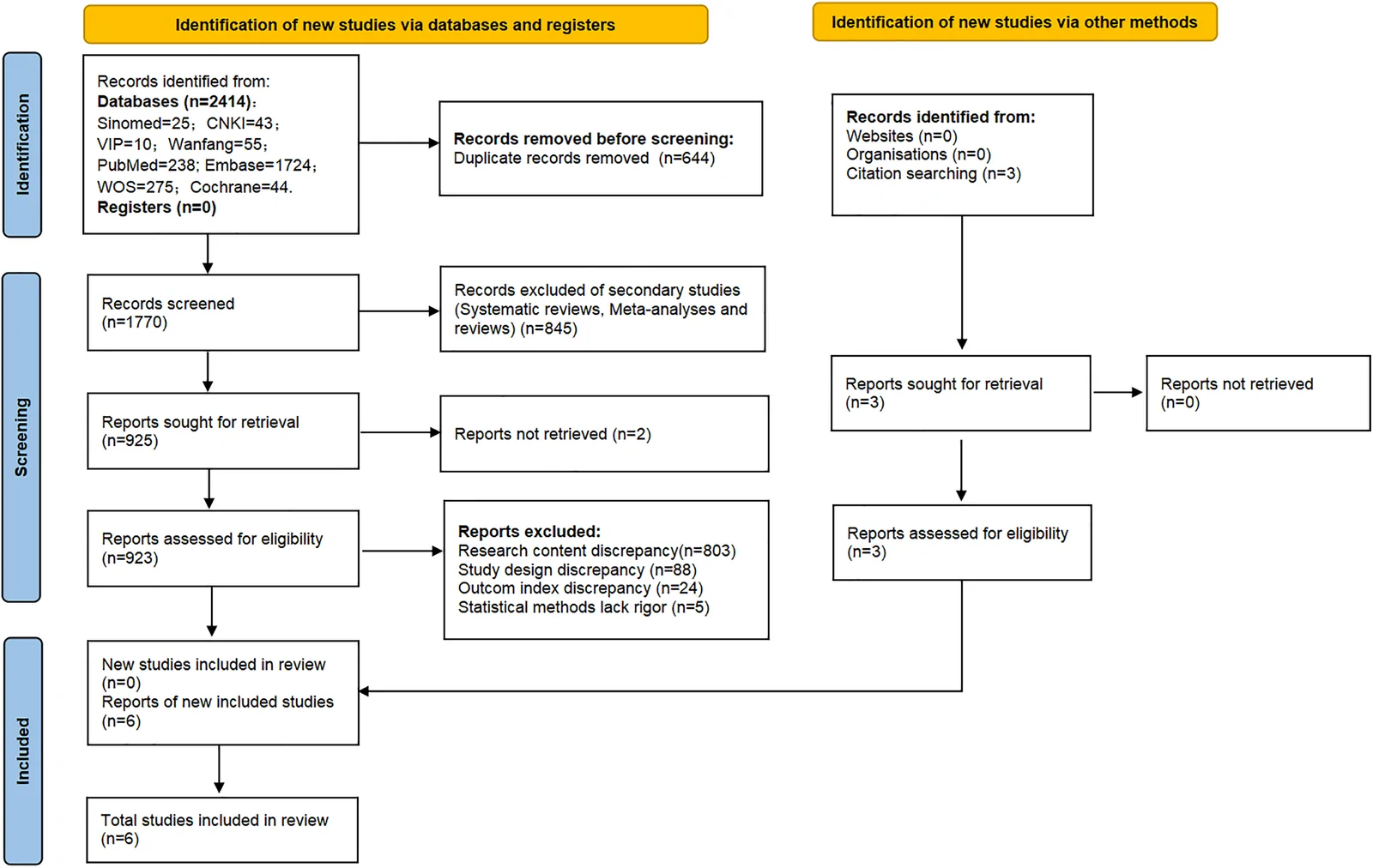
Literature screening flow diagram.

### Description of the included studies

After conducting a thorough literature selection process in line with the PRISMA 2020 standards, six studies published between 2019 and 2026 were identified as meeting the criteria for inclusion in this review. These studies consisted of four retrospective cohort analyses, one prospective cohort analysis, and one case-control study, with participant numbers ranging from 124 to 119,684. In total, they encompassed 131,568 individuals diagnosed with sepsis or septic shock from various locations, including China, Taiwan, and the United States. Key details of the studies included are outlined in Table 2. The quality assessment results for all studies are detailed in Table 3, while Table 4 lists the various factors linked to stress ulcers and gastrointestinal bleeding, presented as adjusted odds ratios (aOR).

**Table 2:** Literature general information.

First Author (year Of Publication)	Location, Time Frame	Study Design	Participants	Sepsis And Septic Shock Diagnostic Criteria	Statistical Methods	Su/Gib Frequency	Population Characteristics	Definition Of Su/Gib
Liangyan Y (2022) [15]	China, 2019–2021	Retrospective Cohort	Sepsis Patients In Ed-Icu, N = 344	Sepsis-3 Criteria; Patients With Septic Shock Were Not Explicitly Excluded Or Separately Reported.	Multivariate Analysis	23.3%	Age 67.5 Yr; Male 67.4%; Su Mortality 26.3% Vs Non-Su 6.4% (p < 0.001)	Stress Ulcer Is Defined As Overt Gastrointestinal Bleeding (e.g., Haematemesis, Melaena), Or Occult Bleeding With A Positive Faecal Occult Blood Test And No Other Identifiable Bleeding Source; Unexplained Haemoglobin Drop >20 G/l; Endoscopic Findings Showing Mucosal Erosion Or Ulceration Predominantly In The Gastric Fundus And Body.
Gang L (2019) [16]	China, 2016–2017	Case-control	Sepsis Patients, N = 133	Sepsis-3 Criteria; Patients With Septic Shock Were Not Explicitly Excluded Or Separately Reported.	Logistic Regression	Not Reported (endoscopy-confirmed Su)	Age 63.7 Yr; Male 49.6%; Multi-organ Dysfunction Focus	Stress Ulcer Status Was Determined Based On Clinical Data And Mods (multiple Organ Dysfunction Syndrome) Scores.
Siddiqui A H (2020) [17]	United States, 2003–2012	Retrospective Cohort	Adult Septic Shock, N = 119684	Icd-9 Codes For Adult Septic Shock; All Participants Had Septic Shock.	Multivariate Analysis	5.4%	Age 70.85 Yr; ↑ Los + 4.8d, ↑ Mortality 54% Vs 45%, ↑ Cost $49,836 (p < 0.001)	Gastrointestinal Bleeding (Gib) Defined By Icd-9 Diagnosis Codes And Corresponding Clinical Classification Software (Ccs) Codes From The Nationwide Inpatient Sample Database.
Xiangdang H (2023) [18]	China, 2020–2023	Retrospective Cohort	Sepsis Patients, N = 124	Sepsis-3 Criteria; Patients With Septic Shock Were Not Explicitly Excluded Or Separately Reported.	Multivariable Logistic Regression	17.7%	Age 67.5 Yr; Male 67.4%; ↑In-hospital Mortality In Gib Group	Upper Gastrointestinal Bleeding Is Defined According To The 2018 Chinese Guidelines For The Diagnosis And Treatment Of Acute Non‑variceal Upper Gastrointestinal Bleeding; Lower Gastrointestinal Bleeding Is Defined According To The 2020 Chinese Guidelines For The Diagnosis And Treatment Of Lower Gastrointestinal Bleeding.
Huang A H (2021) [19]	Taiwan, 2015–2016	Prospective Cohort	Patients With Bloodstream Infection, N = 1,034	Blood Culture-confirmed Bsi With Clinical Signs Of Sepsis Or Acute Systemic Infection; Septic Shock Was Not Specifically Excluded Or Separately Reported.	Multivariable Logistic Regression	7.6%	Age 64.2 ± 17.5 Yr; Male 50.3%; 90-day Mortality Focus	Gastrointestinal Bleeding Defined As: (1) Confirmed By Endoscopy Or Ct Angiography; Or (2) Any Documented Evidence Of Melaena, Haematemesis, Haematochezia, Or “Coffee Grounds” Material In Nasogastric Aspirate During Hospitalisation, Followed By Prescription Of Proton Pump Inhibitors.
Zhang Y (2026) [20]	China, 2016–2023	Retrospective multicenter cohort	Sepsis patients, n = 10,249	Sepsis-3 criteria; septic shock was not explicitly excluded or reported separately.	Multivariable logistic regression	2.0%	Age 70.2 ± 15.8 yr (MGB) vs 66.0 ± 17.0 (non-MGB); Male 66% (MGB) vs 60% (non-MGB); In-hospital mortality 44.0% (MGB) vs 17.0% (non-MGB)	Major gastrointestinal bleeding (MGB) defined as: (1) overt bleeding (haematemesis, melena, haematochezia, or bloody gastric aspirate); (2) haemoglobin drop >20 g/L within 24 h; or (3) transfusion of ≥2 units of packed red blood cells within 24 h in the absence of other bleeding sources.

ED-ICU: emergency department intensive care unit; GIB: gastrointestinal bleeding; ICD-9: International Classification of Diseases, 9th Revision; LOS: length of stay; SIRS: systemic inflammatory response syndrome; SU: stress ulcer; BSI: bloodstream infection. Sepsis-3 criteria: defined as a suspected or confirmed infection accompanied by an acute increase of ≥2 in the Sequential Organ Failure Assessment (SOFA) score relative to baseline.

**Table 3:** Quality assessment of the included studies.

NEWCASTLE – OTTAWA QUALITY ASSESSMENT									
First author (year of publication)	Selection				Comparability	Outcome/Exposure			Total score
	Q1	Q2	Q3	Q4	Q1	Q1	Q2	Q3	
Liangyan Y (2022) [15]	1	1	1	1	2	1	1		8
Gang L (2019) [16]	1	1	1	1	1	1			6
SIDDIQUI A H (2020)^17^	1	1	1	1	2	1	1	1	9
Xiangdang H (2023) [18]	1	1	1	1	2	1	1		8
Huang A H (2021) [19]	1	1	1	1	2	1	1	1	9
Zhang Y (2026) [20]	1	1	1	1	2	1	1	1	9
**COHORT STUDIES**Selection: Q1: Representativeness of the exposed cohort Q2: Selection of the non exposed cohort Q3: Ascertainment of exposure Q4: Demonstration that outcome of interest was not present at start of study Comparability: Q1: Comparability of cohorts on the basis of the design or analysis Outcome: Q1: Assessment of outcome Q2: Was follow-up long enough for outcomes to occur Q3: Adequacy of follow up of cohorts **CASE CONTROL STUDIES** Selection: Q1: Is the case definition adequate? Q2: Representativeness of the cases Q3: Selection of Controls Q4: Definition of Controls Comparability: Q1: Comparability of cases and controls on the basis of the design or analysis Exposure: Q1: Ascertainment of exposure Q2: Same method of ascertainment for cases and controls Q3: Non-Response rate									

**Table 4:** Associated fatcors in included studies.

First Author (year Of Publication)	Associated Factors	AOr	95%Ci
**Liangyan Y (2022)** [15]	Respiratory Failure	3.41	1.60 - 7.24
	Blood Urea Nitrogen (Bun)	1.05	1.00 - 1.09
	Shock	2.43	1.15 - 5.14
**Gang L (2019)** [16]	High Mobility Group Box 1 (Hmgb1)	2.92	2.07 - 4.12
	Elevated Toll‑like Receptor 2 (Tlr2)	2.21	1.72 - 2.84
	Elevated Toll‑like Receptor 4 (Tlr4)	2.18	1.67 - 2.84
**Siddiqui A H (2020)** [17]	Coagulopathy	1.34	1.17 - 1.54
	Transient Ischemic Attack/stroke	1.46	1.17 - 1.83
	Anemia	1.17	1.02 - 1.33
	Mechanical Ventilation	1.45	1.23 - 1.64
	Diabetes	0.78	0.67 - 0.91
	Hypertension	0.84	0.74 - 0.94
	Coronary Artery Disease	0.72	0.60 - 0.86
	Peptic Ulcer Disease	1.71	1.00 - 2.91
	Cirrhosis	1.56	1.12 - 2.17
	Gastroesophageal Reflux Disease	0.57	0.41 - 0.79
**Xiangdang H (2023)** [18]	Previous Anticoagulant Use	6.67	4.17 - 10.65
	History Of Gastrointestinal Bleeding	2.60	2.10 - 3.21
	Mechanical Ventilation > 4 Days	4.06	2.75 - 5.99
	History Of Corticosteroid Use During Hospitalization	4.82	2.45 - 9.47
	Icu Length Of Stay	22.42	7.25 - 69.36
	Platelet Count (Plt)	7.90	3.38 - 18.47
	Prothrombin Time (Pt)	5.94	3.01 - 11.74
	Activated Partial Thromboplastin Time (Aptt)	13.91	4.10 - 47.19
	International Normalized Ratio (Inr)	3.31	2.16 - 5.07
	Apache Ⅱ	64.41	16.21 - 255.87
	Sofa	31.88	8.86 - 114.73
**Huang A H (2021)** [19]	Very Old Age (age ≥ 85)	2.40	1.27 - 4.54
	Hemiparetic Stroke	3.12	1.82 - 5.36
	Hiv	8.90	2.17 - 36.46
	Absence Of Fever	2.18	1.30 - 3.64
	Biliary Tract Infection	1.92	1.03 - 3.56
**Zhang Y (2026)** [20]	Age	1.01	1.00–1.02
	Renal dysfunction	1.61	1.07–2.41
	Malignancy or immunosuppression	1.55	1.12–2.15
	Gastrointestinal infection	1.50	1.11–2.03
	Blood urea nitrogen (BUN)	1.03	1.01–1.04
	Urinary tract infection	0.29	0.16–0.53
	Baseline hemoglobin (HGB)	0.99	0.99–1.00

aOR: adjusted odds ratio; CI: confidence interval; APACHE II: Acute Physiology and Chronic Health Evaluation II; HMGB1: high mobility group box protein B1; HIV: human immunodeficiency virus; SOFA: Sequential Organ Failure Assessment.aOR values represent the risk of stress ulcer associated with each factor after adjusting for potential confounders.

### Associated factors for stress ulcers and gastrointestinal bleeding in patients with sepsis or septic shock

Our results are detailed individually for stress ulcers (SU) and gastrointestinal bleeding (GIB). Within these categories, we classify the associated factors into three domains: those related to the patient, to the disease, and to the treatment.

#### Factors associated with stress ulcer.

A retrospective cohort study conducted at a single center involving 344 patients with sepsis found that respiratory failure (aOR 3.41, 95% CI 1.60–7.24), increased blood urea nitrogen (BUN) levels (aOR 1.05, 95% CI 1.00–1.09), and the presence of shock (aOR 2.43, 95% CI 1.15–5.14) were all significantly linked to a higher odds of SU [15]. Additionally, a case-control study focused on molecular mediators revealed that elevated levels of high mobility group box 1 (HMGB1) in peripheral blood (aOR 2.92, 95% CI 2.07–4.12), as well as increased levels of Toll-like receptor 2 (TLR2) (aOR 2.21, 95% CI 1.72–2.84) and Toll-like receptor 4 (TLR4) (aOR 2.18, 95% CI 1.67–2.84), were each independently associated with a heightened odds of SU among septic patients [16].

#### Factors associated with gastrointestinal bleeding.

**Patient-related factors:** In patients experiencing septic shock, certain pre-existing conditions were found to be linked with gastrointestinal bleeding (GIB). Specifically, peptic ulcer disease (aOR 1.71, 95% CI 1.00–2.91) and cirrhosis (aOR 1.56, 95% CI 1.12–2.17) were linked to a heightened odds, whereas gastroesophageal reflux disease appeared to be associated with a reduced odds (aOR 0.57, 95% CI 0.41–0.79) [17]. Additionally, a history of gastrointestinal hemorrhage was a strong predictor of GIB related to sepsis (aOR 2.60, 95% CI 2.10–3.21) [18]. Factors such as hemiparetic stroke (aOR 3.12, 95% CI 1.82–5.36), advanced age (≥85 years) (aOR 2.40, 95% CI 1.27–4.54), and HIV infection (aOR 8.90, 95% CI 2 [17].–36.46) were also independently associated with an increased odds of acute upper GIB in individuals with bloodstream infections [19]. In a comprehensive multicenter study, the presence of malignancy or immunosuppression (aOR 1.55, 95% CI 1.12–2.15) and advancing age (aOR 1.01, 95% CI 1.00–1.02) were found to elevate the odds of major GIB (MGB) [20]. On the other hand, conditions such as diabetes (aOR 0.78, 95% CI 0.67–0.91), hypertension (aOR 0.84, 95% CI 0.74–0.94), and coronary artery disease (aOR 0.72, 95% CI 0.60–0.86) were associated with a reduced odds of GIB in septic shock patients^17^. Furthermore, a higher initial hemoglobin level was associated with a lower odds of MGB (aOR 0.99, 95% CI 0.99–1.00)^20^, while anemia was linked to an increased odds of GIB (aOR 1.17, 95% CI 1.02–1.33)^17^.

**Disease-related factors:** Markers indicating organ dysfunction were consistently linked to an increased odds of bleeding. In patients experiencing septic shock, transient ischemic attack or stroke (aOR 1.46, 95% CI 1.17–1.83) and coagulopathy (aOR 1.34, 95% CI 1.17–1.54) were found to be independent predictors of gastrointestinal bleeding [17]. Additionally, renal impairment (aOR 1.61, 95% CI 1.07–2.41) and heightened blood urea nitrogen levels (aOR 1.03, 95% CI 1.01–1.04) were independently linked to major gastrointestinal bleeding [20].

Abnormalities in coagulation emerged as significant predictors. In a case-control analysis, factors such as platelet count (aOR 7.90, 95% CI 3.38–18.47), prothrombin time (aOR 5.94, 95% CI 3.01–11.74), activated partial thromboplastin time (aOR 13.91, 95% CI 4.10–47.19), and international normalized ratio (aOR 3.31, 95% CI 2.16–5.07) were found to be independently linked to gastrointestinal bleeding resulting from sepsis [18]. Additionally, disease severity metrics demonstrated strong exponential associations: APACHE II (aOR 64.41, 95% CI 16.21–255.87) and SOFA (aOR 31.88, 95% CI 8.86–114.73) were significantly related to gastrointestinal bleeding due to sepsis^18^.

The source of infection also played a role in the likelihood of bleeding. Specifically, biliary tract infections (aOR 1.92, 95% CI 1.03–3.56) and the lack of fever (aOR 2.18, 95% CI 1.30–3.64) were significant predictors of acute upper gastrointestinal bleeding [19]. Additionally, gastrointestinal infections raised the odds of major gastrointestinal bleeding (aOR 1.50, 95% CI 1.11–2.03), while urinary tract infections appeared to offer a protective effect (aOR 0.29, 95% CI 0.16–0.53) [20].

**Treatment-related factors:** In patients experiencing septic shock, the use of mechanical ventilation was found to have a significant associations with gastrointestinal bleeding (GIB) (aOR 1.45, 95% CI 1.23–1.64) [17], Furthermore, extended mechanical ventilation lasting more than four days was linked to GIB resulting from sepsis (aOR 4.06, 95% CI 2.75–5.99) [18]. The prior administration of anticoagulants (aOR 6.67, 95% CI 4.17–10.65) and the use of corticosteroids during the hospital stay (aOR 4.82, 95% CI 2.45–9.47) were also identified as independent predictors of sepsis-related GIB, with the duration of ICU stay being the most strongly associated factor (aOR 22.42, 95% CI 7.25–69.36) [18].

## Discussion

This systematic review thoroughly examined and combined the elements linked to the presence of stress ulcers (SU) and gastrointestinal bleeding (GIB) in individuals suffering from sepsis or septic shock. It incorporated data from six observational studies involving a total of 131,568 sepsis or septic shock patients. The findings reveal that various factors affect the likelihood of SU/GIB in this group, which can be divided into three categories: patient-specific, disease-specific, and treatment-specific. These results offer crucial insights for recognizing patients at potential elevated risk and for the application of tailored stress ulcer prophylaxis (SUP) approaches in clinical settings.

The analysis revealed a notable association between gastrointestinal bleeding (GIB) and factors such as coagulation disorders, shock, mechanical ventilation, liver dysfunction, and kidney failure in sepsis patients. These results are consistent with the Guidelines for the Prevention of Stress-Related Gastrointestinal Bleeding in Critically Ill Adults, released in 2024 by the Society of Critical Care Medicine (SCCM) and the American Society of Health-System Pharmacists (ASHP) [21]. The guidelines clearly indicate that conditions like coagulopathy, shock, and chronic liver disease heighten the likelihood of stress-related upper gastrointestinal bleeding. They advocate for the use of proton pump inhibitors (PPIs) or histamine-2 receptor antagonists (H2RAs) to mitigate the risk of stress ulcer in critically ill adults facing these risks. However, the guidelines did not identify mechanical ventilation as a distinct factor associated with stress-related upper gastrointestinal bleeding, whereas our review observed a significant association between mechanical ventilation and GIB. This difference may reflect differences in the study populations, as the pathophysiology of sepsis could exacerbate mucosal injury related to mechanical ventilation. Because this review assessed associations rather than independent risk factors, this finding should be interpreted with caution. This difference may arise from the specific characteristics of the study populations, as the pathophysiology of sepsis could exacerbate the mucosal injury caused by mechanical ventilation. Additionally, the 2024 SCCM/ASHP guidelines stress the need to discontinue SUP promptly when risk factors are resolved or the critical condition improves, highlighting the necessity of halting SUP before transferring to the ICU to avoid inappropriate prescriptions. This recommendation emphasizes the critical nature of precise risk assessment and ongoing evaluation.

### Stratification of associated factors and clinical implications

This analysis identified factors associated with stress ulcers and gastrointestinal bleeding (GIB), which can be categorized into three domains: patient-related, disease-related, and treatment-related. This categorization offers valuable insights for clinical decision-making.

Patient-related factors can be further divided into non-modifiable and potentially modifiable factors. Non-modifiable factors included older age, history of peptic ulcer disease, history of GIB, liver cirrhosis, HIV infection, and malignancy or immunosuppression. These factors were significantly associated with an increased likelihood of GIB. Potentially modifiable factors included low baseline hemoglobin levels and anemia, which were also associated with a higher likelihood of GIB. This suggests that healthcare providers should focus on evaluating and addressing anemia during patient admission.

Disease-related factors, such as signs of organ failure (coagulation abnormalities, renal dysfunction, and elevated BUN levels) and severity assessment scores (APACHE II and SOFA), were closely associated with GIB. The source of infection also played a role: biliary tract and gastrointestinal infections were associated with a higher likelihood of GIB, whereas urinary tract infection appeared to be associated with a lower likelihood. This indicates that healthcare providers should consider the specific source of infection when evaluating the likelihood of GIB in sepsis patients.

Treatment-related factors, such as prolonged mechanical ventilation (particularly exceeding four days), prior anticoagulant use, glucocorticoid administration during hospitalization, and extended ICU stay, were associated with a higher likelihood of GIB. These factors suggest potential harm to the gastrointestinal mucosa from medical procedures. Consequently, healthcare providers should carefully evaluate the benefits and risks of these treatments and discontinue them as early as feasible.

### Implications for SUP in clinical practice

In recent times, the extensive application of stress ulcer prophylaxis (SUP) has come under significant examination. Research indicates that regular use of SUP may not provide clear advantages for all individuals suffering from sepsis. In fact, it could elevate the likelihood of negative outcomes, including pneumonia acquired during hospitalization. A review conducted by Begović in 2025 highlighted that the SUP-ICU and PEPTIC studies reveal a relationship between the treatment’s effectiveness and the severity of the illness [22]. Some patient subgroups even showed an increase in mortality rates. Additionally, the overuse of SUP in intensive care units and its application beyond the suggested duration are prevalent concerns [23]. In light of this, it has become crucial to accurately identify patients at high risk and to adopt tailored SUP approaches.

The various associated factors highlighted in this analysis support the need for a precision-based strategy, but they should be further assessed in future prospective cohort studies to determine whether they are predictive or risk factors for stress ulcers and gastrointestinal bleeding in patients with sepsis or septic shock. Healthcare providers can perform thorough evaluations of risks tailored to individual patient situations, taking into account severity scores and laboratory results. This may enable more accurate decisions regarding SUP, pending validation of these factors in prospective studies.

### Integrated pathophysiological perspective

Mucosal damage in sepsis involves multiple interrelated pathways. One major contributor to gastric mucosal ischemia is hemodynamic instability, which is evidenced by shock and insufficient organ perfusion, such as elevated BUN levels [24]. Coagulopathy, characterized by conditions like thrombocytopenia and extended aPTT, has been associated with an increased likelihood of upper gastrointestinal bleeding (UGIB), reflecting its impact on bleeding events during critical illness [25,26]. Furthermore, sepsis is linked to an increased likelihood of upper gastrointestinal bleeding (GIB) [27]. In addition to these traditional factors, our comprehensive analysis points to the possible role of dysregulated inflammation. The strong association between severity scores (APACHE II and SOFA) and the likelihood of SU emphasizes the importance of illness severity. Moreover, the association between unusual clinical presentations, such as afebrile bacteremia, and bleeding events indicates that a modified host response may be related to greater mucosal susceptibility.

One key takeaway from this review is that therapeutic interventions have a notable influence could be associated with SU. Factors such as mechanical ventilation, anticoagulation therapy, and extended use of corticosteroids were associated with a higher likelihood of SU/GIB in the included studies. Interestingly, a prolonged stay in the ICU itself presents a significantly high odds ratio, acting as a composite indicator of cumulative iatrogenic effects. This aspect of iatrogenesis contributes to the ongoing discussion surrounding SUP. While earlier foundational research and meta-analyses suggested that SUP (using H2RAs or PPIs) decreases the incidence of clinically significant gastrointestinal bleeding (GIB) in high-risk individuals [28], the overall advantages of SUP are now under scrutiny. Recent extensive cohort studies focusing on septic patients at risk for GIB have shown no meaningful differences in mortality, bleeding occurrences, or pneumonia rates between those who received SUP and those who did not [29]. Additionally, some findings indicate that PPIs might be linked to increased mortality and bleeding risks compared to H2RAs in septic patients, challenging previous network meta-analyses that supported PPIs for preventing bleeding [28,30]. These inconsistencies underscore a significant gap in understanding: the impact of SUP may vary in the highly inflammatory environment of sepsis compared to the broader ICU patient population.

### Limitations

This analysis presents several limitations. A major limitation is that the included observational studies assessed associations rather than formally distinguishing risk factors from predictive factors. Therefore, we could not determine whether the identified factors were true risk factors or predictive factors for stress ulcers and GIB in patients with sepsis or septic shock. In addition, the studies reviewed were few in number, with a predominance of retrospective designs. The absence of extensive, prospective research could undermine the strength of the findings. The criteria for diagnosing SU and GIB also differed among the studies, such variability may compromise the dependability of the outcomes. Furthermore, certain studies failed to sufficiently account for confounding variables. Although most studies provided adjusted odds ratios (ORs), the variables selected for adjustment were inconsistent, potentially impacting the comparability of the findings. A quantitative meta-analysis was not performed, which means pooled effect sizes for specific associated factors could not be established. Finally, the majority of the studies originated from China, Taiwan, and the United States, with limited representation from other areas such as Europe and Africa, which may restrict the applicability of the findings.

### Future research directions

In light of this review’s conclusions, subsequent studies should focus on several key areas. Firstly, there is a need for more prospective, multicenter research involving larger participant groups to confirm the identified associated factors and yield more accurate estimates of effect sizes. Secondly, a risk prediction model for stress ulcers specifically designed for the sepsis population should be created. This model ought to incorporate various predictive factors to facilitate ongoing risk evaluation and minimize the occurrence of gastrointestinal bleeding. Thirdly, randomized controlled trials are essential to assess the benefit-risk balance of risk-stratified approaches compared to traditional SUP methods in sepsis patients. Lastly, additional exploration into the molecular processes that lead to stress ulcers and gastrointestinal bleeding in sepsis is crucial for discovering new treatment options.

## Conclusions

This comprehensive review reveals that the likelihood of stress ulcer (SU) and gastrointestinal bleeding (GIB) in individuals with sepsis or septic shock may be shaped by a complex interaction of factors related to the patient, the disease, and the treatment. Key factors consistently associated with these outcomes included disorders of coagulation, the presence of shock, the need for mechanical ventilation, as well as liver and kidney dysfunction, the severity of the disease, and previous occurrences of GIB. However, the included studies assessed associations rather than formally distinguishing risk factors from predictive factors. Therefore, these results highlight the need for further prospective cohort studies to determine whether these associated factors are true risk factors or predictive factors for SU and GIB in patients with sepsis or septic shock. Such research is essential before tailored preventive strategies for stress ulcer prophylaxis (SUP) can be designed and recommended. Amidst increasing doubts regarding the blanket application of SUP strategies, this review provides a summary of factors associated with SU and GIB, but does not offer evidence-based guidance for clinical choices. Nonetheless, additional high-quality research is essential to validate these identified associated factors and inform the development of targeted preventive measures.

## Notes

All data are in the manuscript; any additional questions should be addressed to the lead author(s).
